# Pathogenic bacteria in biogas plants using cattle, swine, and poultry manure

**DOI:** 10.17221/47/2024-VETMED

**Published:** 2025-05-23

**Authors:** Ladislav Cermak, Eva Pechouckova, Milan Marounek, Tereza Paulova

**Affiliations:** ^1^Institute of Animal Science, Department of Physiology of Nutrition and Quality of Animal Products, Prague, Czech Republic; ^2^Department of Microbiology, Nutrition and Dietetics, Faculty of Agrobiology, Food and Natural Resources, Czech University of Life Sciences, Prague, Czech Republic

**Keywords:** anaerobic digestion, animal slurry, bacterial pathogens, fugate

## Abstract

Fugate, a waste product from biogas production, regularly used in agriculture as a fertiliser, may contain bacterial pathogens that cause zoonoses. Anaerobic digestion (AD) can inactivate viable pathogens, including parasites, viruses, and pathogens containing antibiotic resistance genes. This study aimed to compare the numbers of pathogenic bacteria and diversity of potential bacterial pathogens in the fugate using three different types of slurry: cattle, swine, and poultry manure. The swine fugate showed higher numbers of *Clostridium perfringens* and *Campylobacter* sp. than the poultry and cattle fugate. In the cattle fugate, the lowest total number of pathogenic bacteria and a low number of coliforms were detected after the AD. The use of cattle manure in biogas plants presents a lower potential for soil contamination with pathogens. The fugate produced using poultry or swine manure can be used carefully to avoid possibility of contamination of aquifers or surface waters. Also fugate produced from manure of cows suffering from chronic botulism can be used only with carefulness because of the presence of *Clostridium botulinum* spores in biogas waste of diseased cows.

Anaerobic digestion (AD) is a complex process in which microbial activity gradually decomposes organic matter in slurry into a mixture of metabolites, gases (biogas), and the residues of organic matter (digestate).

Slurry is the suspension of solids in water that remains after the digestion of feeds in the animal’s digestive tract. The slurry composition in ruminants and non-ruminants differs. The non-gaseous product of AD of a slurry consists of the fugate, a liquid portion, and the digestate (or separate), a solid portion. The production of biogas represents a source of renewable energy (methane), and can be used to replace fossil fuels (Weiland 2010; Wiater and Horysz 2017). The manure is rich in ammonia and low in energy. This imbalance can be improved using maize silage. Slurry and crops grown for energy are mostly used together in agricultural biogas processing plants (BPP), and maize silage has the largest share. The reasons why maize is the most widely used for anaerobic fermentation are its high yield potential, the favourable quality characteristics, and the possibility of preserving the mass by ensiling (Ayhan et al. 2013). The co-digestion of manure with other organic residues offers several advantages, including enhanced loading of biodegradable organic matter, dilution of toxic substances, improved buffer capacity, higher biogas yield, superior digested product quality, and cost reduction (Zhang et al. 2013; Borowski et al. 2014).

The origin of the slurry affects the composition of the bacterial community in the fermenter. The digestate, i.e., the residue after anaerobic fermentation, is a valuable organic fertiliser with a good availability of nitrogen (Rolka et al. 2024), which is comparable to manure in its use. Alternatively, a solid fraction can be separated from the digestate (the separate), which can then be composted, used as bedding, or dried and incinerated. The liquid residue, the fugate, is usually applied to arable land or permanent grassland, or, according to the technology, it is returned to the fermentation process in the biogas processing plant (Kolar et al. 2010). Pathogenic microorganisms in digestate are bacteria such as Salmonella and other microorganisms that may present a health risk for people and animals (Sahlstrom 2003; Sahlstrom et al. 2008). AD can inactivate viable pathogens, such as parasites, viruses, and pathogens containing antibiotic resistance genes (Kearney et al. 1993; Manser et al. 2015). This is important for using digestate as a fertiliser (Koszel and Lorencowicz 2015). The mechanism of pathogen inactivation has not yet been fully elucidated, however, the temperature, acidity, and the presence of antimicrobials are important. In a study with strains of *Salmonella, Shigella, Escherichia coli,* and *Proteus,* volatile fatty acids at concentrations of 70–120 mmol/l inhibited bacterial multiplication within the pH range of 6.0–6.5 (Prohaszka and Baron 1982).

Our experiment aimed to determine the numbers and diversity of pathogenic bacteria of different groups isolated in the fugate from biogas plants using cattle, swine, and poultry manure. The effect of the time of AD (early or late stage) on the numbers of pathogens and total anaerobes was determined, too.

## MATERIAL AND METHODS

### Sampling

Samples of fugates were obtained from selected agricultural biogas plants (BP) in the Central Bohemia and Pilsen regions of Czechia. The BPs were a convenience sample, located within driving distance, and whose operators agreed to the sampling. The authors considered the BP’s chosen to be representative of BPs in this region of the country. Selected BPs belong to large agricultural plants and breed animals with high performance. The pig farm breeds three-breed hybrids, the dairy farm had Holstein cows, and the poultry farm used Hisex Brown hens. Maize hybrids are widely used for silage production. Feed concentrates were prepared from local components. Wheat was supplemented with xylanase. The poultry diet composition conformed to the NRC (1994). Samplings were carried out in three separate BPs, each of which used manure from cattle, swine, or poultry as the main input material for biogas production. Every BP processed only one type of manure per sampling period. There were 30 samples in total (5 samples of the digestate, i.e., early samples, and 5 samples of the fugate after the completed anaerobic digestion process, i.e., late AD in each selected BP). It was not possible to obtain all early and late-stage AD samples on the same day; thus, the BPs processing different types of slurry were sampled on three different days. Furthermore, we had to consider the opinion of the staff of AD operators.

All samples were cultured for all 4 bacteria. Immediately after sampling from the fermenter outlet valve, the samples were placed in sterile sealable sample bottles to the laboratory, where they were processed for microbial cultivation on the same day.

### Cultivation

Campylobacters, clostridia, salmonella, coliform bacteria, and total anaerobes were cultured directly under specific conditions. Salmonella grew on X.L.D. agar (CM0469) and coliforms on MacConkey agar No. 3 (CM0115) under aerobic conditions at 37 °C for 24 hours. Clostridia were cultured on a Perfringens Agar Base TSC & SFP (CM0587), supplemented with Egg Yolk Emulsion (SR0047) and TSC Supplement (SR0088) in an anaerostat under anaerobic conditions for 24 h at 37*** °***C. Campylobacter was cultured on Campylobacter Selective Agar (CM0689) using CampyGen (CN0025), supplemented with Preston Campylobacter Selective Supplement (SR0117) and Laked Horse Blood (SR0048) for 72 h at 37*** °***C. Total anaerobes were grown anaerobically on Wilkins-Chalgren Agar (CM0619) for 24 h at 37 °C. A ten-fold serial dilution of each sample in peptone water containing 10 g bacterial peptone (LP0037B) and 5 g NaCl per 1 litre of distilled water was done, and 50 μl was spread on the respective agar plates. CFU/g dry matter numbers were converted to log CFU/g and subjected to statistical analysis. All media and accessories were supplied by OXOID CZ, s.r.o. (Brno, Czech Republic), and the OXOID catalogue numbers are given in parentheses.

The results were expressed as the mean and the standard deviation.

### Statistical calculation

The *t*-test was used to evaluate the effect of anaerobic digestion on the number of bacteria. The effect of slurry origin on the number of bacteria was analysed using the one-way analysis of variance (Graph PAD Software v1.14; Birthe Avery, MD, USA). Differences were considered significant at *P* < 0.05.

## RESULTS

The swine fugate at late AD showed the highest number of all bacterial types, with low numbers of coliforms after the late AD process (Table 1).

**Table 1:** The table shows the numbers (log CFU) of cultured bacteria in digestate (early AD samples) and BP-derived fugate (late AD samples) using different slurry: *Clostridium perfringens*, Campylobacters, coliform bacteria, and total anaerobes in early and late stages of anaerobic digestion

Bacteria	Stage of AD	Cattle			Swine			Poultry	
		CFU g^–1^	SD		CFU g^–1^	SD		CFU g^–1^	SD
*C. perfringens*	early	5.67	0.28		7.32	0.19		5.83	0.09
	late	5.70^a^	0.14		7.49^b^	0.31		6.64^ab#^	0.10
									
*Campylobacter* sp.	early	5.00	0.63		4.55	0.03		4.49	0.40
	late	4.43^a^	0.01		5.40^a^	0.66		4.99^a^	0.52
									
Coliforms	early	5.39	0.13		5.08	0.30		4.44	0.15
	late	4.43^a#^	0.01		5.38^a#^	0.41		5.13^a^	0.17
									
Anaerobes	early	7.85	0.28		8.29	0.11		7.47	0.32
	late	7.65^a^	0.34		8.40^a^	0.26		7.65^a^	0.33

Presented values are means ± SD; Five samples of digestate were taken from each BP processing one type of slurry, and five samples of fugate after the completed AD, i.e., 30 samples in total; Each sample was cultured for all 4 bacteria

^a,b^Values in the same row with different superscripts differ significantly (*P* < 0.05); ^#^Significant effect of anaerobic digestion (*P* < 0.05) when comparing means for early vs late AD for each bacteria–slurry combination

AD = anaerobic digestion; BP = biogas plant; CFU = colony forming units

The lowest numbers of *Clostridium perfringens, Campylobacter* sp., and coliforms were observed at the late stage of AD in the biogas plant using cattle manure. Low numbers of total anaerobic bacteria were at the late stage of AD from biogas plants using cattle and poultry manure (7.65 log CFU/g). The effect of the time of mesophilic digestion (about two weeks) was significant in BP using swine and poultry manure (an increase of pathogens in the late AD).

Contrary to this, in biogas plants using cattle manure, the number of coliforms decreased significantly during the anaerobic digestion. This implies that the effect of anaerobic digestion on pathogenic bacteria differed in different experimental groups. Coliform bacteria at the late stage were found in similar CFU at the late stage of AD from plants using swine and poultry slurry (log CFU = 5.38 and 5.13, respectively). Salmonella was detected in the poultry samples only, but at the detection limit (data not shown). Significantly, most anaerobes were found in swine fugate late-stage AD samples (log CFU = 8.4 after digestion) compared to cattle and poultry fugate (log CFU = 7.65 both).

## DISCUSSION

The EU regulations on animal by-products stipulate acceptable bacterial levels for digestate and fugate leaving biogas plants for use in agriculture, such as for fertiliser. The current acceptable levels can be found in Commission Regulations on the use of animal by-products and derived products not intended for human consumption (European Commission 2011). It should be emphasised that even if the level of indicator pathogens is below the detection limit, this cannot be directly considered as the absence of a potential pathogenic risk due to the presence of other pathogens.

The study results showed significant differences in the numbers of monitored bacteria groups between samples from biogas plants processing different types of slurry. The swine fugate showed higher numbers of *Clostridium perfringens* and *Campylobacter* sp. than those from poultry and cattle fugate. The lowest number of pathogens was detected in the cattle fugate, with a low number of coliforms after the anaerobic digestion process.

Our results show that the use of cattle manure has the least impact on soil contamination by pathogens. Thus, the use of cattle manure appears to be the most acceptable in terms of protecting human and livestock health. In a future experiment, the survival of pathogenic bacteria present in fugate applied on arable land or permanent grassland should be evaluated. The effect of anaerobic digestion on pathogenic bacteria was not unequivocal. Bacteria differ in susceptibility to pH, temperature, shortage of nutrients, and presence of antimicrobials.

Considering the purpose of anaerobic digestion effluent usage as biofertiliser in agriculture, it is important to highlight that such effluents are suitable and accepted only if the sanitary safety is satisfactorily guaranteed. Increased attention should be given to coliform bacteria and *Clostridium* spp. Coliform bacteria belong to the family *Enterobacteriaceae*. This family includes numerous pathogens, e.g., *Salmonella* sp., *Shigella* sp., and enterotoxigenic species of *Escherichia coli* (Zhang et al. 2023). *Enterobacteriaceae* occur in human and animal intestines and are recognised as being able to survive and multiply under a wide range of stress conditions and hostile environments due to their high tolerance to variation of temperature and pH (Fisher and Phillips 2009; da Costa et al. 2013). In one study, enterococci and *Enterobacteriaceae* seemed predominant in digestate products obtained from mesophilic AD (Bagge et al. 2005). The composition of microbial communities in different systems may differ due to manure handling practices and content, and environmental conditions (Sahlstrom 2003).

Froschle et al. (2015) investigated the incidence of clostridia in 154 plant and animal substrates, digester sludges, and digestates. Findings indicated minor relevance of clostridial pathogens; however, *Cl.* *difficile* was often detected. Also, other pathogenic clostridia can be detected in biogas plants. Biogas plant waste thus can represent a biohazard risk of clostridia for humans and animals. Neuhaus et al. (2015) also examined 24 liquid manure and 84 biogas waste samples from dairies where most cows suffered from chronic botulism. The occurrence of *Cl.* *botulinum* spores in biogas waste of diseased cows indicates an increased and underestimated health risk. The application of digestate as fertiliser could be risky considering the long lifespan of spores in the environment.
